# Alberta’s Tomorrow Project: adherence to cancer prevention recommendations pertaining to diet, physical activity and body size

**DOI:** 10.1017/S1368980016003451

**Published:** 2017-01-25

**Authors:** Heather K Whelan, Jian-Yi Xu, Sanaz Vaseghi, Geraldine Lo Siou, S Elizabeth McGregor, Paula J Robson

**Affiliations:** 1 Alberta’s Tomorrow Project, CancerControl Alberta, Alberta Health Services, Richmond Road Diagnostic and Treatment Centre, 1820 Richmond Road SW, Calgary, AB, Canada, T2T 5C7; 2 Department of Community Health and Epidemiology, College of Medicine, University of Saskatchewan, Saskatoon, SK, Canada; 3 Population, Public and Aboriginal Health, Alberta Health Services, Calgary, AB, Canada; 4 Alberta’s Tomorrow Project, CancerControl Alberta, Alberta Health Services, Edmonton, AB, Canada

**Keywords:** Cancer prevention guidelines, Cancer risk factor, Cohort study, Health promotion, Lifestyle

## Abstract

**Objective:**

To explore cross-sectional adherence to cancer prevention recommendations by adults enrolled in a prospective cohort in Alberta, Canada.

**Design:**

Questionnaire data were used to construct a composite cancer prevention adherence score for each participant, based on selected personal recommendations published by the World Cancer Research Fund/American Institute for Cancer Research (2007). Data were self-reported on health and lifestyle, past-year physical activity and past-year FFQ. The scores accounted for physical activity, dietary supplement use, body size, and intakes of alcohol, fruit, vegetables and red meat. Tobacco exposure was also included. Scores ranged from 0 (least adherent) to 7 (most adherent).

**Setting:**

Alberta’s Tomorrow Project; a research platform based on a prospective cohort.

**Subjects:**

Adult men and women (*n* 24 988) aged 35–69 years recruited by random digit dialling and enrolled in Alberta’s Tomorrow Project between 2001 and 2009.

**Results:**

Of the cohort, 14 % achieved adherence scores ≥5 and 60 % had scores ≤3. Overall adherence scores were higher in women (mean (sd): 3·4 (1·1)) than in men (3·0 (1·2)). The extent of overall adherence was also associated with level of education, employment status, annual household income, personal history of chronic disease, family history of chronic disease and age.

**Conclusions:**

Reported adherence to selected personal recommendations for cancer prevention was low in this cohort of adults. In the short to medium term, these results suggest that more work is required to identify behaviours to target with cancer prevention strategies at a population level. Future work will explore the associations between adherence scores and cancer risk in this cohort.

Cancer causes 8·2 million deaths each year worldwide, accounting for 13 % of all deaths. Annual numbers of new cancer cases worldwide are expected to rise by 70 % over the next two decades^(^ ^1^ ^)^.

While greater uptake of screening and increasingly effective treatments have reduced mortality rates for some cancers^(^ ^2^ ^–^ ^4^ ^)^, there is a growing realization that primary prevention through lifestyle and environmental interventions may offer a more sustainable solution for reducing cancer burden^(^ ^1^ ^,^ ^2^ ^,^ ^5^ ^)^.

Estimates for the impact of modifiable risk factors on cancer incidence vary according to the assumptions used to create the statistical models and on the source and quality of the underlying data^(^ ^6^ ^,^ ^7^ ^)^. However, regardless of the variation in different population-attributable fractions for each risk factor, there is a general consensus that use of tobacco, physical inactivity, low fruit and vegetable intake, high intake of red and processed meats, overweight and obesity, and use of alcohol are responsible for up to 30 % of cancer incidence overall^(^ ^8^ ^–^ ^13^ ^)^.

In 2007, the World Cancer Research Fund/American Institute for Cancer Research (WCRF/AICR) produced a series of population goals and personal recommendations for cancer prevention based on these modifiable behaviours and health-related variables^(^ ^14^ ^)^. The rationale was to provide a robust evidence base to inform the development of policies and interventions to help reduce cancer risk in the general adult population.

However, before moving towards policy development, it is important to understand more about the concurrence between existing health-related variables and behaviours and current recommendations. Such information may be useful in identifying priority areas for further investigation and intervention.

The aim of the current study was to explore the extent of adherence to WCRF/AICR cancer prevention recommendations for individuals^(^ ^14^ ^)^, based on behaviours and health-related variables reported by adults enrolled in Alberta’s Tomorrow Project (ATP), Alberta, Canada. A comprehensive overview of adherence to cancer prevention guidelines and the associated influential factors can inform cancer prevention strategies and assist in designing consistent targeted strategies to increase population adherence to evidence-based cancer prevention guidelines.

## Methods

### Study population

Between 2001 and 2009, ATP enrolled 31 212 Albertans aged 35–69 years, with no personal history of cancer other than non-melanoma skin cancer, into a cohort. Participants were recruited by random digit dialling using Regional Health Authority boundaries as the sampling frame. In the first recruitment wave, a second eligible adult within each household was recruited (*n* 382, 1 %) if possible; but this practice was discontinued in subsequent recruitment waves^(^ ^15^ ^)^.

Recruits were mailed an information package and were considered enrolled if they returned a completed Health and Lifestyle Questionnaire (HLQ) and signed consent form. Three months after enrolment, participants received two additional questionnaires assessing past-year dietary habits and physical activity. Response rate to the random digit dialling call has been estimated at 32 %^(^ ^15^ ^)^ and response rate of those individuals to enrolment was 49 %.

### Questionnaires

The HLQ queried self-reported sociodemographic, behavioural and health-related variables including age, marital status, education, employment status, annual household income, tobacco use and exposure to second-hand smoke, family health history and body measurements (height, weight, waist and hip circumferences). In addition, participants indicated whether or not a doctor had ever told them that they had any of the following health conditions: high blood pressure, angina, high blood cholesterol, heart attack, stroke, emphysema, chronic bronchitis, diabetes, ulcerative colitis, Crohn’s disease, hepatitis or liver cirrhosis. Participants who answered ‘yes’ to having received a diagnosis of any of these conditions were deemed to have a personal history of chronic disease.

Past-year diet, including supplement use, was assessed by the US National Cancer Institute’s 124-item Canadian Diet History Questionnaire I (CDHQ-I), an FFQ adapted for use in Canada^(^ ^16^ ^,^ ^17^ ^)^. Recent evidence supports that the food list in CDHQ-I is representative of the foods commonly consumed by Canadian adults^(^ ^18^ ^)^. CDHQ-I data were analysed using Diet*Calc version 1.4.3 (National Cancer Institute, Bethesda, MD, USA) to estimate mean daily intakes of nutrients and food group servings, as well as supplement use.

Past-year recreational activities were assessed using a validated Past Year Total Physical Activity Questionnaire (PYTPAQ)^(^ ^19^ ^)^. Total minutes per week performing leisure activities at moderate (3–6 MET, where MET=metabolic equivalent of task) and vigorous (>6 MET) intensities were calculated based on reported activities.

### Cancer prevention recommendations adherence score

A composite score reflecting adherence of reported behaviours and health-related variables to seven selected WCRF/AICR personal recommendations for cancer prevention was constructed for each participant using data collected upon enrolment to ATP. Recommendations included were those that addressed the general adult population and were identified as quantifiable using baseline ATP data. Recommendations addressing special groups (cancer survivors, breast-feeding women, people living in specific geographic regions) or that were not quantifiable with ATP data were excluded. It was not possible to quantify adherence to some recommendations (consume fast foods sparingly, avoid sugary drinks, limit sedentary habits), while the data required to quantify some other recommendations were not available for ATP participants (consume energy-dense foods sparingly, limit refined starchy foods, eat relatively unprocessed grains and legumes, avoid salt-preserved, salted and salty foods, do not eat mouldy cereals and pulses, avoid weight gain and increases in waist circumference throughout adulthood, ensure body weight through childhood and adolescent growth projects towards the lower end of the normal BMI range). Considering the WCRF/AICR panel emphasized the importance of tobacco exposure for cancer risk^(^ ^14^ ^)^ in addition to the other recommendations, second-hand smoke exposure and cigarette smoking were also included in the composite score. Participants scored 1 point for each recommendation met and 0 if it was not met. Recommendations included in the current analyses were: BMI within normal range (18·5–24·9 kg/m^2^), no daily exposure to tobacco during the past year, no more than two alcoholic drinks daily for men or no more than one daily for women, consumption of five or more servings of fruit and vegetables daily, consumption of less than 500 g of red meat weekly, not taking any dietary supplements and performing at least 210 min of moderate- or vigorous-intensity recreational physical activity weekly. The composite score ranged from 0 (least adherent) to 7 (most adherent).

### Statistical analyses

Descriptive statistics were presented as group means and standard deviations for continuous variables and as counts and percentages for categorical variables. Multiple linear regression models were used to explore associations between participants’ composite scores and potential influential characteristics. Multiple logistic regression models were used to explore the association between participants’ adherence to each of the seven components of the composite score and potential influential factors. All estimation models were adjusted for age (at categorical level: 35–49, 50–59, 60–70 years), marital status (living without partner, living with partner), education level (high school or lower, college, university), employment status (not employed, retired, employed part-time, employed full-time), annual household income (<$CAN 70 000, ≥$CAN 70 000), first-degree family history of cancer (no, yes), first-degree family history of chronic disease (no, yes) and personal history of chronic disease (no, yes). Analyses of the associations were reported as adjusted regression coefficients for continuous outcomes and adjusted odds ratios for binary outcomes, and corresponding 95 % confidence intervals.

All analyses were stratified by sex and the criterion for statistical significance was set as *α*≤0·05 (two-tailed). All analyses were performed using the statistical software package SAS version 9.2.

## Results

Of 31 212 participants enrolled in ATP, the following were excluded from the current analyses: second in household recruit (*n* 382), outside age range of 35–69 years at enrolment (*n* 46), pregnant women (*n* 65), BMI<18·5 kg/m^2^ (*n* 220, may indicate pre-existing disease), personal history of cancer other than non-melanoma skin cancer prior to enrolment (*n* 38), not living in Alberta at enrolment (*n* 61) and biologically implausible energy intake of <3347 or >17 572 kJ/d (<800 or >4200 kcal/d) for men and <2510 or >14 644 kJ/d (<600 or >3500 kcal/d) for women (*n* 1014)^(^ ^20^ ^)^. Participants were also excluded if their log-transformed total energy expenditure derived from the PYTPAQ fell outside two interquartile ranges from the first and third quartile cut-offs^(^ ^21^ ^,^ ^22^ ^)^ (*n* 92). Participants who did not return the CDHQ-I or PYTPAQ (*n* 4212), participants with incomplete data for BMI (*n* 22), smoking status (*n* 17), and past-year second-hand smoke exposure (*n* 55) were also excluded, resulting in a final sample of 24 988.

Among all participants, 37 % were men (mean age 51·1 (sd 9·1) years) and 63 % were women (mean age 50·9 (sd 9·2) years). All other baseline sociodemographic characteristics are presented in Table 1.

**Table 1:** Baseline characteristics reported by Alberta’s Tomorrow Project participants, Canada, stratified by sex

		All participants		Men		Women	
Baseline characteristic	Category	Frequency	%*	Frequency	%*	Frequency	%*
Age (years)							
	≥35 and <50	12 233	49·0	4424	47·8	7809	49·6
	≥50 and <60	7819	31·3	2977	32·2	4842	30·8
	≥60 and <70	4936	19·7	1852	20·0	3084	19·6
Marital status†							
	Living without partner	5285	21·2	1524	16·5	3761	23·9
	Living with partner	19 700	78·8	7728	83·5	11 972	76·1
Education level‡							
	High school or lower	6879	27·5	2265	24·5	4614	29·3
	College	9884	39·6	3743	40·4	6141	39·0
	University	8224	32·9	3245	35·1	4979	31·7
Employment status§							
	Not employed	3393	13·6	496	5·4	2897	18·4
	Retired	3397	13·6	1187	12·8	2210	14·0
	Employed part-time	4248	17·0	604	6·5	3644	23·2
	Employed full-time	13 942	55·8	6963	75·3	6979	44·4
Annual household income ($CAN)							
	<70 000	12 187	49·9	3949	43·3	8238	53·9
	≥70 000	12 221	50·1	5172	56·7	7049	46·1
First-degree family history of cancer║							
	Yes	13 354	53·4	4729	51·1	8625	54·8
First-degree family history of chronic disease¶							
	Yes	14 432	57·8	5056	54·6	9376	59·6
Personal history of chronic disease**							
	Yes	11 280	45·1	4537	49·1	6743	42·9

* Column percentages.

† Living without partner=divorced, separated, widowed or single (never married); living with partner=married, or not married but living with someone.

‡ High school or lower=did not complete Grade 8, completed Grade 8 but not high school, completed high school; college=some technical school/college training completed, completed technical school/college training; university=some part of university degree completed, completed university degree, some part of postgraduate university degree completed, completed university postgraduate degree.

§ Not employed=not employed but looking for work, homemaker and student; retired=retired; employed part-time=less than 30 h/week; employed full-time=30 h or more/week.

║ Yes=if any one of father, mother, full-blooded brothers, full-blooded sisters, sons or daughters of the participant had been diagnosed with cancer of the breast, ovary, rectum, colon, prostate or any other type of cancer; otherwise ‘no’.

¶ Yes=if any one of father, mother, full-blooded brothers, full-blooded sisters, sons or daughters of the participant had been diagnosed with heart attack, stroke or diabetes; otherwise ‘no’.

** Yes=participant had been told by a doctor that they had one of the following medical conditions: high blood pressure, angina (chest pains from a heart problem), high cholesterol in blood, heart attack, stroke, emphysema, chronic bronchitis, diabetes, ulcerative colitis, Crohn’s disease, hepatitis or liver cirrhosis; otherwise ‘no’.

Approaches for operationalizing the selected WCRF/AICR personal recommendations, and numbers and proportions of participants whose reported behaviours or health-related variables complied with those recommendations, are presented in Table 2. Adherence to the selected WCFR/AICR personal recommendations was highest for alcohol consumption (88 %), while adherence to the tobacco exposure recommendation was lowest (15 %). Forty-eight per cent of participants met the recommended amount of physical activity per week. A greater proportion of men (29 %) than women (14 %) reported that they did not use dietary supplements. Conversely, greater proportions of women than men reported behaviours that adhered to recommendations for body size (40 % *v*. 23 %), consumption of fruits and vegetables (44 % *v*. 35 %) and red meat (89 % *v*. 65 %).

**Table 2:** World Cancer Research Fund/American Institute for Cancer Research (2007) personal recommendations for cancer prevention: operationalization and proportions of Alberta’s Tomorrow Project participants meeting recommendations

					
Personal recommendations	Operationalization	Scoring	All (%*)	Men (%*)	Women (%*)
Body fatness ∙ Maintain body weight within the normal range from age 21 ∙ Avoid weight gain and increases in waist circumference throughout adulthood	BMI ≥25·0 kg/m^2^	0			
	BMI=18·5–24·9 kg/m^2^	1	33·9	22·9	40·4
	Waist circumference not included in scoring†				
Physical activity ∙ Be moderately physically active, equivalent to brisk walking, at least 30 minutes per day ∙ Aim for 60 minutes or more of moderate activity, or 30 minutes or more of vigorous activity, every day ∙ Limit sedentary habits	<210 min of moderate- or vigorous-intensity‡ recreational physical activity/week over the past 12 months	0			
	≥210 min of moderate- or vigorous-intensity‡ recreational physical activity/week over the past 12 months	1	48·1	51·0	46·3
	Sedentary habits not included in scoring§				
Foods and drinks that promote weight gain ∙ Consume energy-dense foods sparingly ∙ Avoid sugary drinks ∙ Consume fast foods sparingly, if at all	Not included in scoring§				
Plant foods ∙ Eat at least 5 portions/servings (400 g or 14 oz) of a variety of non-starchy vegetables and/or fruits every day ∙ Eat relatively unprocessed cereals (grains) and/or pulses (legumes) with every meal ∙ Limit refined starchy foods ∙ People who consume starchy roots or tubers as staples also to ensure intake of sufficient non-starchy vegetables, fruit and pulses (legumes)	<5 servings of fruit and vegetables/d over the past 12 months║	0			
	≥5 servings of fruit and vegetables/d over the past 12 months║	1	40·6	34·8	43·9
	Refined starchy food and unprocessed grains and legumes not included in scoring§				
Animal foods ∙ Consume less than 500 g (18 oz) of red meat per week, very little if any to be processed	≥500 g red meat/week¶	0			
	<500 g red meat/week¶	1	80·2	64·8	89·3
	Processed meat not included in scoring§				
Alcoholic drinks ∙ If consumed, limit consumption to no more than 2 drinks per day for men and 1 drink per day for women	>2 drinks/d for men and >1 drink/d for women	0			
	≤2 drinks/d for men and ≤1 drink/d for women	1	87·8	87·6	87·9
Food preservation, processing, preparation ∙ Avoid salt-preserved, salted, salty foods. Preserve foods without using salt ∙ Limit consumption of processed food with added salt to ensure an intake of less than 6 g (2·4 g sodium) per day ∙ Do not eat mouldy cereals (grains) or pulses (legumes)	Not included in scoring§				
Dietary supplements ∙ Dietary supplements are not recommended for cancer prevention	At least one dietary supplement taken over the past 12 months**	0			
	No dietary supplement use over the past 12 months**	1	19·6	28·7	14·2
Tobacco exposure ∙ Avoid exposure to tobacco smoke	Exposed to tobacco in the past year††	0			
	Not exposed to tobacco in the past year††	1	14·9	14·2	15·4

* Column percentages.

† Waist circumference highly correlated with BMI (Pearson’s correlation coefficient=0·8389).

‡ Moderate or vigorous recreational physical activity calculated by MET (metabolic equivalent of task), MET≥3 reported in recreation and leisure activities included.

§ The data required were not available for participants or it was not possible to quantify the adherence.

║ Includes tomato and all kinds of green and yellow vegetables. Excludes dry beans and peas, white potato, starchy vegetables, fruit juice and fruit drinks. These numbers are generated by Diet*Calc software based on the FFQ (Canadian Diet History Questionnaire I) data.

¶ Includes beef, lamb and pork; excludes organ meats.

** Dietary supplements include: vitamin A, vitamin B_1_ (thiamin), vitamin B_2_ (riboflavin), vitamin B_3_ (niacin), vitamin B_6_, vitamin B_9_ (folic acid), vitamin B_12_, vitamin C, vitamin D, vitamin E, β-carotene, calcium, magnesium, iron, zinc, copper, selenium.

†† Exposed to tobacco in the past year=current daily smokers (at least one cigarette every day for the past 30 d), current occasional smokers (at least one cigarette in the past 30 d, but not every day), reported being exposed to second-hand smoke in the previous year at home, in a car or other private vehicle, in public places (bars, restaurants, shopping malls, arenas, bingo halls, bowling alleys), when visiting friends or relatives, at work.

**Fig. 1:**
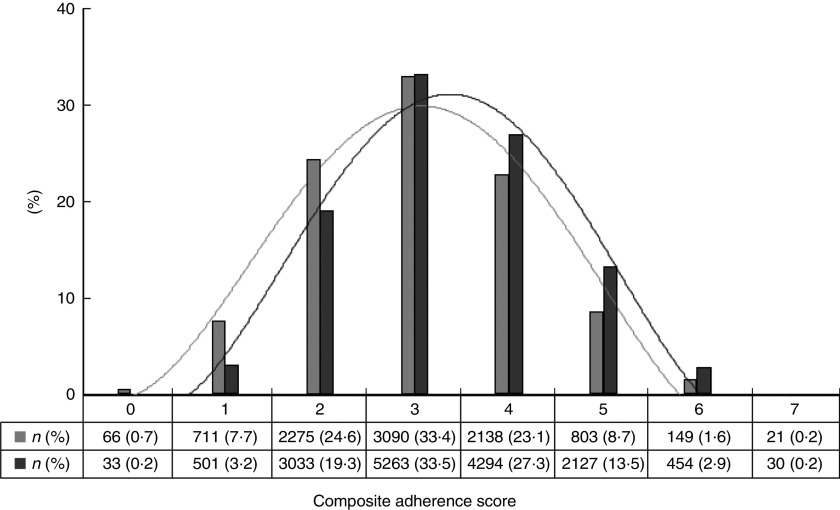
Frequency distribution of composite adherence scores reflecting the extent of participants’ adherence to cancer prevention recommendations, as reported in the Alberta’s Tomorrow Project cohort, Canada, stratified by sex (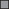, men; 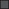, women)

Compiling these data into a composite score demonstrated that overall adherence to selected personal recommendations for cancer prevention was low in this cohort (mean score 3·3 (sd 1·2)). The proportion of participants (60 %) with an adherence score ≤3 was four times greater than the proportion with a composite score ≥5 (14 %). The mean score in women was 3·4 (sd 1·1) and in men was 3·0 (sd 1·2), *P*<0·0001. The frequency distribution of women’s reported adherence to cancer prevention recommendations was more favourable than that observed for men (Fig. 1).

Table 3 presents the associations between participant characteristics and their reported overall adherence to cancer prevention recommendations as indicated by the adherence composite score. In men, reported adherence to selected personal recommendations for cancer prevention was 0·10 (95 % CI 0·02, 0·18) points higher in those aged 60–70 years than in the younger age groups. Compared with all other influential factors, a post-secondary education was associated with the greatest reported overall adherence both in men (0·50 (95 % CI 0·44, 0·57) points higher) and women (0·36 (95 % CI 0·31, 0·41) points higher for university *v*. those who had a high school education or lower). In men, being retired (0·35 (95 % CI 0·22, 0·48) points higher *v*. not employed) and in women, a higher annual household income (0·21 (95 % CI 0·17, 0·25) points higher *v*. lower annual household income), also showed a strong association with higher overall adherence to recommendations. However, the composite score was 0·10 (95 % CI −0·15, 0·05) points lower in women who were employed full-time compared with women who were not employed. Among all the estimated influential factors, a personal history of chronic disease was associated with the lowest overall adherence to cancer prevention recommendations in men (−0·17 (95 % CI −0·22, 0·12) points lower) and women (−0·25 (95 % CI −0·29, 0·22) points lower *v*. no personal history). A family history of chronic disease in women, but not in men, had a significant negative effect on the adherence to these cancer-specific recommendations.

**Table 3:** Association between adherence to cancer prevention recommendations and potential influential factors in Alberta’s Tomorrow Project cohort, Canada, stratified by sex

	Men (*n* 9114)			Women (*n* 15 276)		
Influential factor	*β* coefficient*	95 % CI	*P* value	*β* coefficient*	95 % CI	*P* value
Age (years)						
≥35 and <50	Reference	–	–	Reference	–	–
≥50 and <60	0·02	−0·03, 0·07	0·4484	−0·03	−0·08, 0·01	0·0818
≥60 and <70	0·10	0·02, 0·18	0·0110	0·04	−0·02, 0·10	0·2108
Marital status†						
Living without partner	Reference	–	–	Reference	–	–
Living with partner	0·01	−0·06, 0·06	0·9733	−0·04	−0·09, −0·01	0·0398
Education level‡						
High school or lower	Reference	–	–	Reference	–	–
College	0·15	0·09, 0·21	<0·0001	0·12	0·08, 0·16	<0·0001
University	0·50	0·44, 0·57	<0·0001	0·36	0·31, 0·41	<0·0001
Employment status§						
Not employed	Reference	–	–	Reference	–	–
Retired	0·35	0·22, 0·48	<0·0001	0·10	0·03, 0·18	0·0035
Employed part-time	0·13	−0·01, 0·27	0·0607	0·02	−0·03, 0·07	0·4566
Employed full-time	0·11	0·01, 0·22	0·0400	−0·10	−0·15, −0·05	<0·0001
Annual household income ($CAN)						
<70 000	Reference	–	–	Reference	–	–
≥70 000	0·06	0·01, 0·11	0·0126	0·21	0·17, 0·25	<0·0001
First-degree family history of cancer║						
No	Reference	–	–	Reference	–	–
Yes	−0·05	−0·09, −0·01	0·0341	−0·03	−0·07, −0·01	0·0449
First-degree family history of chronic disease¶						
No	Reference	–	–	Reference	–	–
Yes	−0·01	−0·06, 0·03	0·5530	−0·06	−0·10, −0·02	0·0005
Personal history of chronic disease**						
No	Reference	–	–	Reference	–	–
Yes	−0·17	−0·22, −0·12	<0·0001	−0·25	−0·29, −0·22	<0·0001

* Estimations for each factor were adjusted for age, marital status, education, employment, annual household income, first-degree family history of cancer, first-degree family history of chronic disease and personal history of chronic disease, except the major independent variable.

† Living without partner=divorced, separated, widowed or single (never married); living with partner=married, or not married but living with someone.

‡ High school or lower=did not complete Grade 8, completed Grade 8 but not high school, completed high school; college=some technical school/college training completed, completed technical school/college training; university=some part of university degree completed, completed university degree, some part of postgraduate university degree completed, completed university postgraduate degree.

§ Not employed=not employed but looking for work, homemaker and student; retired=retired; employed part-time=less than 30 h/week; employed full-time=30 h or more/week.

║ Yes=if any one of father, mother, full-blooded brothers, full-blooded sisters, sons or daughters of the participant had been diagnosed with cancer of the breast, ovary, rectum, colon, prostate or any other type of cancer; otherwise ‘no’.

¶ Yes=if any one of father, mother, full-blooded brothers, full-blooded sisters, sons or daughters of the participant had been diagnosed with heart attack, stroke or diabetes; otherwise ‘no’.

** Yes=participant had been told by a doctor that they had one of the following medical conditions: high blood pressure, angina (chest pains from a heart problem), high cholesterol in blood, heart attack, stroke, emphysema, chronic bronchitis, diabetes, ulcerative colitis, Crohn’s disease, hepatitis or liver cirrhosis; otherwise ‘no’.

**Fig. 2:**
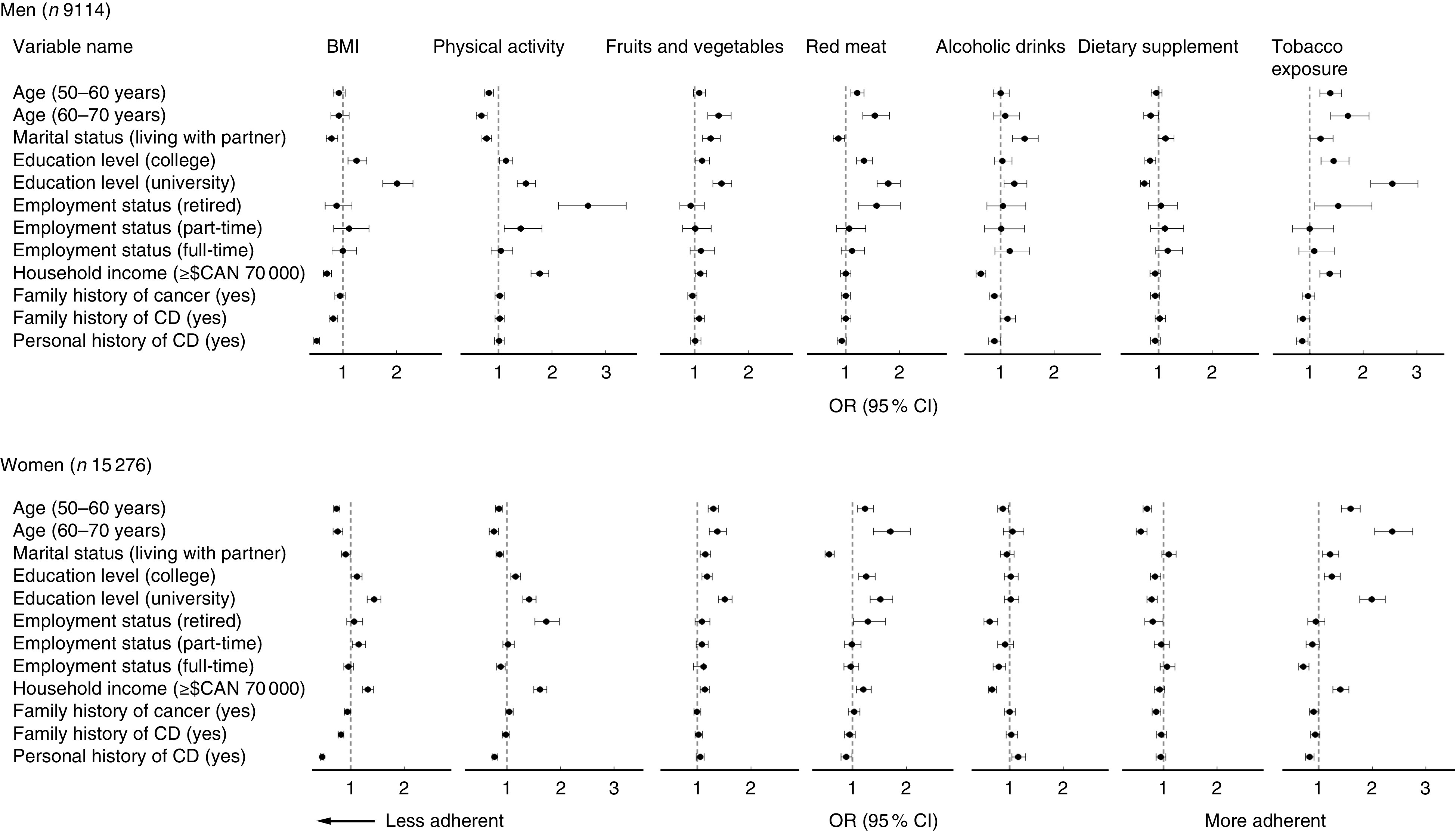
Association between adherence to individual cancer prevention recommendations and influential factors in the Alberta’s Tomorrow Project cohort, Canada, stratified by sex. Values are odds ratios, with their 95 % confidence intervals represented by horizontal bars, adjusted for age, marital status, education level, employment status, annual household income, first-degree family history of cancer, first-degree family history of chronic disease (CD) and personal history of CD. The reference levels for estimated variables are: age (≥35 to <50 years), marital status (living without partner), education level (high school education or lower), employment status (not employed), annual household income (<$CAN 70 000), first-degree family history of cancer (no), first-degree family history of CD (no) and personal history of CD (no)

Figure 2 presents the direction and magnitude of the associations between participant characteristics and reported adherence to each of the seven components included in the composite score.

Higher educational attainment was associated with greater odds of adhering to recommendations for body size. A personal history of chronic disease and a family history of chronic disease were associated with lower odds of adherence to the BMI recommendation. Older women (≥50 years) and men living with a partner were less likely to adhere to the BMI recommendation. In women, an annual household income of ≥$CAN 70 000 was associated with higher odds of meeting the body size recommendation, while in men the association was in the opposite direction.

A personal history of chronic disease was associated with lower odds of adhering to the physical activity recommendation, but only in women. In men, the odds of adhering to the physical activity recommendation were higher in those who were retired or working part-time relative to men who reported not being employed. In women, adherence was higher only in those who were retired. In men and women, adherence to the physical activity recommendation was lower in participants aged ≥50 years and those who were married or living with a partner. Adherence to the physical activity recommendation was higher in people with a post-secondary education and an annual household income ≥$CAN 70 000.

Examination of reported adherence to the fruits and vegetables recommendation demonstrated higher odds of adherence in those who were in older age groups, those who lived with a partner and those who had a post-secondary education. In women, adherence to the fruits and vegetables consumption recommendation was also higher in people with a higher annual household income.

Adherence to the recommendation for red meat intake was influenced by age, marital status and educational attainment. Older age and post-secondary education were associated with greater odds of adhering to the recommendation for red meat intake. Conversely, those who lived with a partner had lower odds of adhering to the red meat recommendation.

Higher household income in men and women was associated with lower odds of adhering to recommendations for alcohol consumption. Relative to women who reported no current employment, women who were retired or worked full-time had lower odds of adhering to the alcohol recommendation. Men living with a partner or who had a university education and women with a personal history of chronic disease had higher odds of adhering to the alcohol consumption recommendation.

Adherence to the recommendation for dietary supplement use was lower in participants with a post-secondary education. In women, adherence was lower in those aged ≥50 years and with a family history of cancer.

Adherence to the tobacco exposure recommendation was likely to be higher in participants aged ≥50 years, with a post-secondary education and an annual household income ≥$CAN 70 000. Participants with a personal history of chronic disease were less likely to adhere to the tobacco exposure recommendation, as were women working full-time.

## Discussion

Over the past two decades, several organizations have published a series of recommendations that aim to provide guidance on strategies for cancer risk reduction based on modifiable behavioural risk factors. Most focus on the themes of achieving and maintaining a body size within the normal range for BMI, eating lower amounts of red meat, being physically active, not consuming alcohol (or consuming low amounts), and consuming diets that are predominantly plant-based and/or relatively high in fruits and vegetables^(^ ^14^ ^,^ ^23^ ^–^ ^28^ ^)^.

In the current study, we aimed to examine the extent to which participants enrolled in a longitudinal cohort reported behaviours that adhered to selected personal recommendations described in the 2007 WCRF/AICR report^(^ ^14^ ^)^. However, in common with previous studies^(^ ^29^ ^–^ ^33^ ^)^, we encountered several challenges in determining and applying cut-offs that could be used to determine adherence to recommendations. For example, ambiguity in the language used by the WCRF/AICR 2007 recommendations resulted in an inability to operationalize the recommendation (e.g. ‘limit refined starchy foods’) because it was not possible to quantify the term ‘limit’. In other cases, we made assumptions or used a proxy indicator. Furthermore, the recommendation to ‘maintain body weight within the normal range from age 21’ was operationalized as BMI within the normal range at the time of completing the enrolment HLQ. Although the body fatness recommendation in the WCRF/AICR report contains three personal recommendations, the other two (‘ensure that body weight through childhood and adolescent growth projects towards the lower end of the normal BMI range at age 21’ and ‘avoid weight gain and increases in waist circumference throughout childhood’) could not be included due to a lack of data pertaining to lifetime body weight and waist circumference of the cohort participants. Others may have chosen different approaches to quantification and, as such, it is difficult to compare our findings directly with other studies. The WCRF/AICR panel did acknowledge the challenges of quantification assessing adherence to recommendations when they included phrases such as ‘limit’ or ‘consume sparingly’, but noted that it is not always possible to establish clear cut-off points^(^ ^14^ ^)^. Others have suggested that there is a clear need for agencies that set guidelines or recommendations to work more closely with researchers and organizations responsible for population health surveillance to ensure that behaviours are well defined and that adherence can be assessed in ways that are practical, feasible, meaningful and also comparable across different populations^(^ ^34^ ^)^.

Despite these challenges, we did identify seven reported behaviours and health-related variables that could be operationalized and used to create a composite score for each individual to indicate adherence. Overall adherence was higher in women than men. These observations are similar to those reported by the European Prospective Investigation into Cancer and Nutrition (EPIC) study, which also examined concordance with WCRF/AICR 2007 recommendations in 386 355 men and women across nine European countries^(^ ^33^ ^)^. Other studies that have calculated adherence scores have reported that adherence to cancer prevention guidelines may reduce overall cancer risk^(^ ^31^ ^)^, reduce postmenopausal breast cancer risk^(^ ^35^ ^)^ and reduce overall risks of cancer, as well as breast and colon cancers^(^ ^36^ ^)^. Conversely, a recent study from the Framingham Offspring Cohort reported no significant associations between overall adherence scores and risk of obesity-related cancers^(^ ^37^ ^)^. However, in the latter study, the sample size was relatively small (*n* 2983) and therefore may have had insufficient power to detect the effects of composite long-term exposure on cancer risk.

One challenge with the composite score approach is that it gives equal weight to all recommendations included in the score^(^ ^38^ ^)^, even though some may not be associated with risk of cancer at specific sites^(^ ^33^ ^)^. More robust estimates of the associations between composite scores and cancer risk may be obtained if different weights were applied to different elements included in such scores. However, the complexities associated with such a task are not to be underestimated. Future work should attempt to include the determination of appropriate weighting for scoring individual items in the assessment of individualized risk.

When each element of the composite score was examined separately, it was clear that some recommendations were more easily adhered to than others. In the ATP cohort, the lowest level of reported adherence was with the tobacco exposure recommendation. Although 15 % of participants were current smokers, 85 % were exposed to tobacco. It should be noted that the majority of participants completed the baseline HLQ prior to the Alberta provincial legislation banning smoking in workplaces coming into effect in 2008. Previous research has indicated that legislative changes in tobacco regulation have decreased exposure^(^ ^39^ ^)^, so the proportion of ATP participants exposed to second-hand smoke may decrease in future follow-up questionnaires.

Adherence to the dietary supplement recommendation was also very low (20 %). This is consistent with the extensive use of supplements (70 %) previously reported in ATP participants^(^ ^40^ ^)^. Some dietary supplements have beneficial effects for health or long-term health conditions, such as neural tube defects^(^ ^41^ ^)^, iron-deficiency anaemia^(^ ^42^ ^)^ and osteoporosis^(^ ^43^ ^)^; however, some supplements could increase cancer risk^(^ ^44^ ^,^ ^45^ ^)^. The CDHQ-I data on supplement use do not provide any information on participant motivation for taking dietary supplements. The WCRF/AICR recommendation is that dietary supplements not be used for cancer prevention, which provides the general public with inconsistent messages when compared with recommendations for the prevention and/or treatment of other conditions.

Adherence to the recommendation to maintain body weight in the normal range was also low. In the ATP cohort, 77 % of men and 60 % of women reported heights and weights that put them in the overweight and obese categories for BMI. Although the physiological mechanisms linking body size with cancer risk are not clear, the WCRF/AICR graded the evidence linking obesity and risks of cancers of the pancreas, colorectum, breast (postmenopause), endometrium and kidney as convincing, suggesting that continued efforts to prevent or reduce the prevalence of obesity in the population should be explored more vigorously as means of helping reduce overall cancer risk and risk of specific cancers.

The highest adherence reported in the present study was to the recommendation for alcohol consumption. The WCRF/AICR report^(^ ^14^ ^)^ states that ‘if alcoholic drinks are consumed, limit consumption to no more than two drinks a day for men and one drink a day for women’. This recommendation is based on the assumption that alcohol may have a cardioprotective effect. However, the evidence linking alcohol consumption with increased cancer risk remains convincing, consistent with the fact that ethanol has been classified as a class I carcinogen^(^ ^46^ ^)^, and supports a recommendation of zero alcohol consumption for cancer prevention. Only 15 % of participants reported consuming no alcohol in the past year, which is consistent with Canadian data concerning alcohol use^(^ ^47^ ^)^. J/U-shaped relationships between alcohol intake and all-cause mortality have been reported^(^ ^48^ ^,^ ^49^ ^)^, again providing inconsistent messaging between preventive behaviour recommendations for different diseases.

Adherence to the recommendation for physical activity was 48 %. This observation is consistent with other reports that Canadian adults are inactive and do not participate in sufficient activity to benefit health^(^ ^50^ ^)^. One challenge in understanding the effects of activity on health is that there is little consensus on how to assess activity in free-living people. Previous research on a subgroup of the ATP cohort has demonstrated relatively low levels of leisure-time activity compared with occupational and household activities^(^ ^51^ ^)^, and it has also been reported previously that ATP participants take part in insufficient leisure-time activity for cancer risk reduction^(^ ^52^ ^)^. Although the personal recommendation does not specify types of activity, the WCRF/AICR Panel did note that all forms of physical activity protect against some cancers. As dose–response to exercise is investigated further, it may be determined that levels of physical activity required for cancer prevention may be different from levels required for CVD and obesity prevention. Since evidence is emerging to suggest that overall activity energy expenditure and sedentary time as independent factors may both be important for cancer and chronic disease risk reduction^(^ ^53^ ^–^ ^56^ ^)^, it will be necessary to work towards identifying and applying consistent approaches to assessing and reporting the exposure.

Strengths of the present study include its large sample size that increased statistical power and provided the potential for subgroup analysis. However, due to the cross-sectional nature of the study, it is not yet possible to draw conclusions about the impact of adherence to cancer prevention guidelines on subsequent cancer incidence. The prospective design of the ATP will allow for the development of a longitudinal data structure to explore the associations in future research.

Limitations of the present study include the participant bias of responses in self-reported questionnaires. In addition, it is recognized that this cohort does not represent the entire population of Alberta, and it has been suggested that those who choose to enrol in a prospective cohort may be more health conscious than the general population^(^ ^57^ ^–^ ^59^ ^)^. However, reporting of descriptive characteristics of the cohort demonstrates that ATP participants represent a diverse cross-section of baseline demographic and behavioural characteristics, with very few differences in general characteristics from the Canadian Community Health Survey Cycle 3.1^(^ ^60^ ^)^, therefore suggesting that the cohort represents a broad range of the population^(^ ^61^ ^)^. The relatively low adherence to the selected recommendations is particularly concerning when longitudinal cohort participants are typically expected to be more health conscious than the general population^(^ ^57^ ^–^ ^59^ ^)^. The operationalization of the adherence composite score presented some challenges and resulted in the exclusion of some of the WCRF/AICR recommendations. The use of typical epidemiological assessment tools is challenging in the assessment of adherence to some of the WCRF/AICR recommendations. Future longitudinal work may be able to address the recommendations pertaining to changes over time and incorporate them into cancer risk models.

## Conclusion

The overall adherence to current cancer prevention guidelines in the cohort was low. Specific areas with low adherence were identified, such as body size and tobacco exposure, suggesting that these may be targets for intervention. Future work that attempts to weight the importance of individual recommendations may identify additional intervention targets that could have more impact in cancer risk reduction, despite potentially having better adherence to recommendations overall. Population health work should stress the need for specific, targeted messaging provided by health-care resources.
